# The Irish Matrons' Association and the Martyr Nurse

**Published:** 1915-11-13

**Authors:** Harriet E. Reed

**Affiliations:** Hon. Sec. Irish Matrons' Association.


### The Irish Matrons' Association and the Martyr Nurse.

*To the Editor of* THE HOSPITAL.

SIR,—At a meeting of the Irish Matrons' Association held at 34 St. Stephen's Green, Dublin, on November 6, the following resolution of sympathy with Mrs. Cavell was proposed by Miss Helen Shuter, seconded by Miss Carson, and passed:—

"We, the members of the Irish Matrons' Association, wish to offer, on behalf of the institutions we represent and the profession to which we belong, our profound sympathy for the grievous loss sustained by Mrs. Cavell, but also to give some expression to the deep pride felt by the whole nursing profession in the splendid example of patriotism, courage, and patience which Miss Cavell has left to it as an inheritance for ever."—Yours truly, HARRIET E. REED,

Hon. Sec. Irish Matrons' Association.

